# Enhanced *H. pylori* Treatment Using a *β*‐Cyclodextrin–Propolis Complex: A Randomized Clinical Trial

**DOI:** 10.1155/bmri/6257147

**Published:** 2026-07-07

**Authors:** Aisa Mehri, Shabnam Pourmoslemi, Alireza Khalilian, Mahdi Safari, Erfan Ayubi

**Affiliations:** ^1^ Department of Pharmaceutics, School of Pharmacy, Hamadan University of Medical Sciences, Hamadan, Iran, umsha.ac.ir; ^2^ Department of Gastroenterology and Hepatology, School of Medicine, Hamadan University of Medical Sciences, Hamadan, Iran, umsha.ac.ir; ^3^ Clinical Research Development Unit of Shahid Beheshti Hospital, Hamadan University of Medical Sciences, Hamadan, Iran, umsha.ac.ir; ^4^ Social Determinants of Health Research Center, Institute of Health Sciences and Technologies, Hamadan University of Medical Sciences, Hamadan, Iran, umsha.ac.ir

**Keywords:** *β*-cyclodextrin, antibiotic resistance, drug delivery, *Helicobacter pylori*, propolis

## Abstract

**Background:**

*Helicobacter pylori* infection poses a significant global health burden, with rising antibiotic resistance and treatment‐related side effects highlighting the need for adjunctive therapies. Propolis, a natural resin with known antimicrobial and anti‐inflammatory properties, suffers from poor solubility and bioavailability. This study evaluated a *β*‐cyclodextrin–propolis complex designed to enhance delivery and therapeutic efficacy in *H. pylori*‐infected patients.

**Materials and Methods:**

In a double‐blind, randomized clinical trial at Hamadan University of Medical Sciences, biopsy‐confirmed *H. pylori* patients received standard quadruple therapy plus omeprazole. The treatment group (*n* = 28) also received 250 mg/day of the *β*‐cyclodextrin–propolis complex for 2 months; the control group (*n* = 12) received placebo capsules. Outcomes were assessed using fecal antigen tests and gastrointestinal symptom questionnaires 2 months posttreatment. Characterization of the complex was performed using FESEM, FTIR, and drug release profiling in PBS (pH 7.4).

**Results:**

The complex demonstrated a successful formation and a sustained propolis release profile. Clinically, 57.1% of treated patients achieved negative antigen tests and symptom resolution, compared with 41.6% in the control group. Adverse effects were mild and comparable between groups.

**Conclusion:**

These findings suggest that the *β*‐cyclodextrin–propolis complex may serve as an effective natural adjunct to standard *H. pylori* treatment, offering potential benefits in eradication rates and symptom relief.

**Trial Registration:**

Iranian Registry of Clinical Trials (IRCT): 20230503058065N1.

## 1. Introduction

Despite global efforts to reduce its burden, *Helicobacter pylori* remains highly prevalent, affecting approximately 4.4 billion people worldwide—about 56% of the global population as of 2022, with the highest infection rates reported in Africa, South America, and parts of Asia [1]. Standard treatment regimens, typically consisting of proton pump inhibitors and multiple antibiotics, are increasingly challenged by growing antibiotic resistance, poor patient compliance, and adverse drug reactions, contributing to declining eradication rates globally [2, 3].

In this context, natural compounds with antimicrobial and gastroprotective properties have gained attention as potential adjuncts to standard treatment protocols. Propolis, a resinous substance collected by honeybees from plant sources, has long been recognized for its broad‐spectrum biological activities, including antibacterial, antiviral, antifungal, anti‐inflammatory, antioxidant, and wound‐healing properties [4–6]. The chemical composition of propolis is complex and varies by geography, but it is generally rich in flavonoids, phenolic acids, and esters, which contribute to its antimicrobial potency against pathogens such as *H. pylori*. However, the clinical use of propolis is limited by its poor water solubility, instability in aqueous environments, and low oral bioavailability [7].

To overcome these limitations, beta‐cyclodextrin (*β*‐CD)—a cyclic oligosaccharide with a hydrophilic outer surface and a hydrophobic cavity—has been widely used as a carrier to enhance the solubility, stability, and bioavailability of lipophilic compounds like propolis [8]. The encapsulation of propolis within *β*‐CD forms an inclusion complex, which improves its gastrointestinal absorption and protects its active components from degradation, thus potentially enhancing its therapeutic efficacy [9]. Preclinical studies have shown that propolis–CD complexes exhibit superior antimicrobial, antineoplastic, anti‐inflammatory, and antioxidant activity compared with raw propolis extract [10–12].

This randomized, double‐blind clinical trial was designed to assess the efficacy and safety of a modified quadruple therapy regimen supplemented with a daily dose of a propolis–*β*‐CD complex in comparison with placebo in patients with confirmed *H. pylori* infection. The primary endpoint was the rate of microbiological eradication of *H. pylori*, as determined by stool antigen testing, whereas secondary outcomes included improvement in gastrointestinal symptoms and the incidence of treatment‐related adverse events. By integrating a natural, bioactive agent with enhanced delivery characteristics into a conventional eradication protocol, this study aims to explore a novel approach for improving therapeutic outcomes in the management of *H. pylori* infection.

## 2. Materials and Methods

### 2.1. Experimental Design

The sample size for this double‐blind randomized clinical trial was calculated based on data from a previous study by Imani et al. [13]., which reported a 26.5% UBT positivity rate in the cinnamon extract group versus 46.9% in the control group. Anticipating that the current intervention would yield at least a 5% greater efficacy than cinnamon extract and assuming a 25% difference between groups, a sample size was calculated to achieve 80% statistical power at a 5% significance level. This resulted in a minimum of 50 participants per group (IRCT [Iranian Registry of Clinical Trials] code: 20230503058065N1; Ethics code: IR.UMSHA.IREC.1402.088).

Participants meeting the inclusion criteria were randomized using block randomization (25 blocks of four participants) to ensure balanced group assignment. Randomization was conducted using a simple random number table. To maintain blinding, neither participants, healthcare providers, nor data collectors were aware of group assignments.

Eligible subjects were adults aged 18–80 years, referred to gastroenterology clinics in Hamadan with gastrointestinal complaints such as dyspepsia, nausea, vomiting, or epigastric pain. A confirmed *H. pylori* infection via gastric biopsy was required for enrollment. Exclusion criteria included a history of gastrointestinal bleeding, serious comorbidities (hepatic, renal, or cardiovascular), known drug allergies, recent use of NSAIDs, corticosteroids, antibiotics, or acid‐suppressing drugs, and alcohol dependence. Participants were also withdrawn if they experienced serious adverse events, failed to comply with the protocol, used nonstudy medications, became pregnant, or voluntarily discontinued participation.

### 2.2. Preparation of *β*‐CD Complexes With Propolis

Iranian propolis was collected between August and September 2023 from the mountainous region of Taleghan in North Iran. Samples were stored in well‐closed containers at 4°C, and for extraction, they were ground to a fine powder by an electric mill. Extraction was performed in ethanol 70% for 72 h on a shaker at room temperature. The extract was filtered, and ethanol was removed using a rotary evaporator (Büchi, Switzerland). The extract was then dried in a 50°C oven and stored in well‐closed containers at 4°C until use.

**Figure 1:**
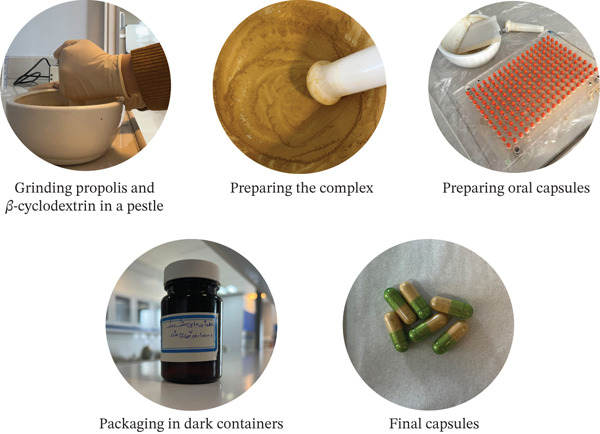
The workflow of preparing the propolis–cyclodextrin inclusion complex.

Propolis and *β*‐CD (food grade, PharmaGrade, China) inclusion complexes were prepared using a solvent‐free physical mixing method, which is considered an environmentally friendly and green chemistry approach [14]. Equal weights of *β*‐CD and propolis were accurately weighed and blended in a ceramic mortar. Due to the low melting point and sticky nature of propolis, the grinding process was performed under controlled temperature and humidity (20°C, 35%, respectively) to ensure uniform mixing and prevent degradation (Figure 1).

The mixture was ground continuously for approximately 40 min to promote intimate contact between the molecules, facilitating the formation of stable inclusion complexes. This mechanical method also reduced the particle size of the components, thereby enhancing the surface area and the potential for interaction between the bioactive compounds of propolis and the cyclodextrin host. The final powdered complex was stored in airtight, light‐ and moisture‐resistant glass containers at 25°C to preserve its stability and biological activity.

### 2.3. Characterization of Propolis–*β*‐CD Complexes

To evaluate the physicochemical characteristics of the synthesized propolis–*β*‐CD inclusion complexes, multiple analytical techniques were employed to assess their structural features, particle morphology, and chemical interactions.

### 2.4. Morphological and Particle Size Analysis

Field Emission Scanning Electron Microscopy (FESEM) was used to investigate the surface morphology and particle size distribution of the freeze‐dried propolis–*β*‐CD complexes. Dried samples were prepared and sent to the Mahamax Research Laboratory (Tehran, Iran) for imaging. Images were captured at appropriate accelerating voltages to visualize the structural integration of propolis within the *β*‐CD matrix. The FESEM analysis enabled comparison of the surface textures of raw materials (propolis and *β*‐CD) with that of the final inclusion complexes, offering insights into particle aggregation, encapsulation uniformity, and changes in surface topology resulting from complex formation. Dynamic light scattering (DLS) was also used to determine the size and polydispersity of the complex particles (Malvern, United States).

### 2.5. Chemical Interaction Analysis

Fourier transform infrared spectroscopy (FTIR) was used to assess molecular interactions and confirm the formation of inclusion complexes between propolis and *β*‐CD. Samples were mixed with potassium bromide (KBr), pressed into pellets, and analyzed across the 400–4000 cm^−1^ range. Spectral comparisons revealed shifts in key absorption bands, indicating noncovalent interactions—such as hydrogen bonding and hydrophobic inclusion—confirming successful complexation between propolis and *β*‐CD.

### 2.6. In Vitro Release Profile Determination of Propolis From Microcapsules

To evaluate the release profile of propolis from the prepared microcapsules, an in vitro release study was performed under simulated physiological conditions. A calibration curve was first established using various concentrations of propolis extract in phosphate‐buffered saline (PBS, pH 7.4), and absorbance was measured using a UV‐Vis spectrophotometer to quantify propolis concentrations. Microcapsules were accurately weighed and dispersed in PBS containing 1% Tween 80, then placed in dialysis bags (MWCO: 12–14 kDa) and immersed in PBS at 37°C under constant shaking (100 rpm). Samples from the release medium were collected at specific time intervals (10, 20, 30, 40, 50, 60, 120, 180, 240, and 1440 min) and replaced with fresh PBS to maintain consistent volume. The amount of released propolis was determined spectrophotometrically at the *λ*max of propolis, and results were used to plot the cumulative release profile over time. This method enabled precise assessment of the kinetics and extent of propolis release from the microcapsules.

### 2.7. Dissolution Test

To assess the dissolution behavior of the propolis–*β*‐CD complex, in vitro studies were conducted using a USP Type II (paddle) dissolution apparatus at 50 rpm. Two buffer systems were selected to simulate gastrointestinal conditions: PBS (pH 7.4) and acetate buffer (pH 4.5–5.5). Dissolution samples were collected at 10, 20, 30, 40, 50, 60, 120, 180, and 240 min. Each sample was filtered and analyzed using UV‐Vis spectrophotometry to determine the absorbance of released compounds. Concentrations were calculated using a standard calibration curve of propolis and expressed as a percentage of total content. All experiments were performed in duplicate, and mean values were used for data analysis. This test enabled evaluation of the release kinetics of active compounds from the complex compared with pure propolis.

### 2.8. Double‐Blind, Randomized Clinical Trial Procedure

This randomized, double‐blind clinical trial evaluated the efficacy and safety of a propolis–*β*‐CD complex as an adjunct to a modified four‐drug regimen for *H. pylori* treatment. Capsules containing 250 mg of the dried propolis–*β*‐CD complex were prepared using a capsule‐filling machine under hygienic conditions to ensure consistent dosage. Placebo capsules, identical in appearance and weight, were filled with starch powder for the control group to maintain blinding. Both groups received a two‐week course of bismuth subcitrate (240 mg), amoxicillin (1 g), and metronidazole (500 mg) twice daily, along with omeprazole (20 mg) once daily, followed by omeprazole alone for an additional month. In parallel, the treatment group received one capsule of the complex daily for 2 months, whereas the control group received a placebo capsule. Initial *H. pylori* infection was diagnosed via biopsy, and treatment effectiveness was assessed 2 months later using a stool antigen test and symptom evaluation questionnaire. Baseline gastrointestinal symptoms and possible side effects—including epigastric pain, bloating, nausea, and others—were rated on a 4‐point scale and monitored throughout the study. Patients were regularly followed via phone and in‐person visits, and those who consumed less than 80% of the prescribed medication were excluded from final analysis. This design ensured reliable assessment of the complex′s therapeutic potential alongside standard treatment.

### 2.9. Statistical Analysis

Data were collected using a researcher‐completed checklist and analyzed with SPSS V17. Descriptive statistics, chi‐square, fisher′s exact, and *t*‐tests were used for normally distributed data, whereas the Mann–Whitney *U* test was applied to nonparametric data. A significance level of 0.05 was set.

## 3. Results

### 3.1. Morphological Analysis of the Cyclodextrin–Propolis Complex

**Figure 2:**
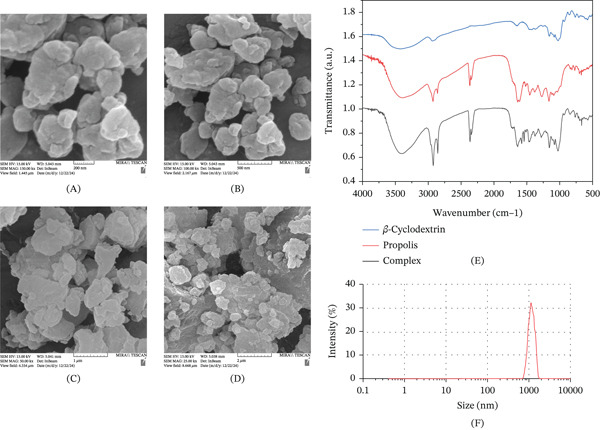
Field Emission Scanning Electron Microscopy (FESEM) images showing surface morphology of the sample at different scales of (A) 200 nm, (B) 500 nm, (C) 1 *μ*m, and (D) 2 *μ*m, respectively. (E) Fourier‐transform infrared (FTIR) spectra confirming the functional groups and chemical interactions present in the complex. (F) Diagram of particles size.

To thoroughly investigate the morphology and particle size of the complexes formed between propolis and *β*‐CD, imaging using a FESEM was employed. The images obtained at various magnifications—200 nm, 500 nm, 1 *μ*m, and 2 *μ*m—enabled simultaneous assessment of the overall structure, particle distribution, and surface microstructural characteristics of the samples (Figure 2A–D).

Analysis of these images revealed that after the complexation process, propolis particles were uniformly distributed within the *β*‐CD structure. The observed morphology predominantly consisted of spherical particles with an approximate size of 50 nm, mostly located in larger aggregates of 1 *μ*m size. FESEM images showed the particle size distribution was highly homogeneous, with minimal variation in dimensions. These results were further approved by data obtained from DLS that showed 1 *μ*m dispersion with low size polydispersity index of 0.4.

However, due to the natural tendency of the particles to aggregate, some microstructures measuring around half a micron were observed in certain images. These larger formations were primarily attributed to particle adhesion during the drying phase of sample preparation.

### 3.2. Structural Analysis of Propolis and Its Complex

The FTIR spectral analysis revealed important molecular interactions between *β*‐CD and propolis during complex formation. Characteristic absorption bands were identified for each component: *β*‐CD showed a broad O–H stretching band (3300–3500 cm^−1^), C–H stretching around 2900 cm^−1^, and fingerprint peaks below 1500 cm^−1^, indicating its cyclic oligosaccharide structure. Propolis exhibited strong O–H and phenolic group absorption near 3300 cm^−1^, a distinct C=O stretching band at 1700 cm^−1^ due to flavonoids and phenolic acids, and aromatic C=C stretching around 1600 cm^−1^, along with C–O and C–O–C peaks in the 1000–1200 cm^−1^ range (Figure 2E).

Upon forming the propolis–*β*‐CD complex, notable changes occurred in the FTIR spectrum. Shifts and broadening of the O–H band, along with weakened or shifted C=O and C=C bands, suggest the establishment of hydrogen bonds and inclusion of propolis molecules within the cyclodextrin cavity. These spectral modifications confirm successful complexation, highlighting both hydrogen bonding and encapsulation effects that stabilize the inclusion complex.

### 3.3. Release Profile of Propolis

The results of evaluating the release rate of propolis from the *β*‐CD–propolis complex compared with pure propolis in a PBS medium (pH 7.4) indicate that, in the initial stages, the amount of propolis released from the complex was significantly higher than that from pure propolis.

**Figure 3:**
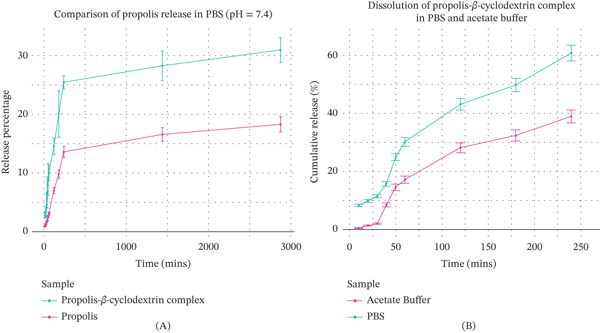
(A) Profile of release of dried propolis extract and propolis–cyclodextrin complex. (B) Profile of dissolution of propolis from propolis–cyclodextrin complex capsules. All tests were performed in three independent experiments and mean amounts were used for generating the diagrams, error bars show SD.

The release study showed that within the first 10 min, only 0.859% of pure propolis was released, whereas 2.75% of the *β*‐CD–complexed propolis was released during the same time frame. This initial difference highlights a faster onset of release from the complex. Over time, the release rate increased for both samples; however, a significant difference in the release pattern was observed (Figure 3A). At 240 min, the release of the complexed propolis reached 25.485%, whereas only 13.612% of pure propolis had been released. At 1440 min (24 h), the complex released 28.32%, in contrast to 16.61% for the pure propolis. By the end of the 48‐h (2880 min) study, the complexed propolis reached a release rate of 31.006%, whereas the pure propolis reached 18.313% (Figure 3A).

These results indicate that complexation of propolis with *β*‐CD enhanced both the amount and rate of release over time. Contrary to the initial assumption that complexation might slow down the release, in this particular case, it not only provided controlled release but also significantly increased the final amount of propolis released. Therefore, it can be concluded that the complexation likely improved the solubility of propolis, thereby enhancing its bioavailability.

### 3.4. Solubility of the Propolis–*β*‐CD Complex

The dissolution study of the propolis–*β*‐CD complex in two media—PBS at pH 7.4 and acetate buffer at acidic pH—demonstrated a significant difference in the release profiles of the active compound. In the neutral PBS environment, the release began rapidly, reaching 8.27% at 10 min and gradually increasing to 60.74% after 240 min. Conversely, in the acidic acetate buffer, the initial release was much slower, with only 0.4% released at 10 min and reaching 38.87% by the end of 4 h (Figure 3B).

These results suggest that the complex maintains better stability and offers more controlled, sustained release under neutral pH conditions, which resemble those of the human gastrointestinal tract. The slower and delayed release in acidic conditions may be influenced by pH‐dependent stability, the chemical structure of *β*‐CD, and interactions with environmental ions. Overall, the findings support the potential of the propolis–*β*‐CD complex to enhance the bioavailability and therapeutic performance of propolis in physiological settings (Figure 3B).

## 4. Clinical Trial

### 4.1. Eradication and Symptom Improvement

In this double‐blind clinical trial designed to evaluate the efficacy of the Propolis–*β*‐CD complex as an adjunct to the standard quadruple therapy regimen for *H. pylori* eradication, 100 eligible patients were randomly assigned to either a treatment group or a control group. The treatment group received one capsule per day containing the propolis complex, whereas the control group was given a placebo consisting of starch. After excluding patients who withdrew or experienced adverse effects, a total of 28 patients in the treatment group and 12 patients in the control group completed the 2‐month course of treatment.

In the treatment group, 16 out of 28 patients (57.1%) achieved full recovery, defined as both resolution of gastrointestinal symptoms and a negative stool antigen test. In contrast, in the control group, only 5 out of 12 patients (41.6%) met the criteria for successful treatment. The remaining patients in both groups either continued to have symptoms and a positive test or reported symptom improvement without undergoing the final test (Table. 1).

**Table 1:** Comparison of *Helicobacter pylori* eradication and symptom improvement between the treatment and control groups.

Final patient outcome	Control group (*n* = 12)	Treatment group (*n* = 28)	Control (%)	Treatment (%)
Complete recovery (negative test + symptom improvement)	5	16	41.60%	57.10%
Symptom improvement but positive test	1	5	8.30%	17.90%
Positive test and persistent symptoms	6	4	50.00%	14.30%
Only reported symptom improvement (no test available)	1	3	8.30%	10.70%
Total	12	28	100%	100%

For statistical analysis and comparison of treatment efficacy between the intervention group (Propolis–*β*‐CD complex) and the control group (placebo), the data were analyzed comprehensively. The primary indicators used to assess efficacy included the results of the stool antigen test (as a marker of gastrointestinal infection or inflammation) and the status of gastrointestinal symptoms (such as abdominal pain, bloating, changes in bowel habits, and other related complaints) after 2 months of follow‐up.

Initially, raw data were organized into a frequency table to determine the distribution of patients with positive or negative test results and those with improved or unchanged symptoms in each group. Subsequently, relative frequencies (percentages) were calculated for each variable to allow comparative analysis between the two groups (Table 2).

**Table 2:** Analysis of treatment success in the treatment and control groups.

Final patient status	Treatment group (*n* = 28)	Control group (*n* = 12)	Treatment %	Control %	*p* (Fisher)
Full recovery (negative test + symptom relief)	16	5	57.10%	41.60%	0.39
Control group (did not achieve full recovery)	12	7	42.90%	58.30%	
Total	28	12	100%	100%	

To assess the statistical significance of the difference in treatment success rates between the intervention (Propolis–*β*‐CD complex) and control (placebo) groups, Fisher′s exact test was applied in Table 3. This statistical test was selected due to the limited sample size in the final groups (28 in the intervention group and 12 in the control group) because the chi‐square test may lack sufficient power and its assumptions may not be met in such conditions. The test result yielded a *p* value of 0.39, indicating that the observed difference in treatment success rates between the two groups was not statistically significant.

**Table 3:** Comparison of the frequency of clinical symptoms and treatment‐related side effects in the control and intervention groups after 2 months.

Adverse event (end of treatment)	Control (*n* = 12)	Intervention (*n* = 28)	Fisher′s exact test *p*
Epigastric pain	4 (33.3%)	5 (17.9%)	0.27
Heartburn	4 (33.3%)	9 (32.1%)	0.93
Abdominal bloating	4 (33.3%)	8 (28.6%)	0.75
Loss of appetite	2 (16.7%)	8 (28.6%)	0.43
Nausea	4 (33.3%)	7 (25.0%)	0.57
Vomiting	1 (8.3%)	1 (3.6%)	0.53
Diarrhea	2 (16.7%)	3 (10.7%)	0.6
Constipation	6 (50.0%)	5 (17.9%)	0.03
Headache	7 (58.3%)	11 (39.3%)	0.23
Metallic taste in mouth	4 (33.3%)	2 (7.1%)	0.04

However, interpreting results based solely on statistical significance may not fully reflect the clinical impact of the treatment. In this study, 57.1% of the remaining patients in the intervention group (16 out of 28) achieved complete recovery, compared with only 41.6% (5 out of 12) in the control group. This relatively notable difference may have clinical relevance—particularly considering that patients who received propolis not only showed a higher rate of negative stool antigen tests but also reported greater improvement in gastrointestinal symptoms, even among those without a negative test. This may indicate a soothing effect of propolis on the gastrointestinal mucosa, which, when combined with cyclodextrin, may have enhanced its bioavailability and therapeutic efficacy.

Moreover, it is important to consider that in studies with small sample sizes, statistical tests may lack the power to detect true differences, increasing the risk of Type II error (*β* error), in which a real difference is mistakenly accepted as null. Another key point is that the combined criterion of symptom improvement alongside a negative test provides a more comprehensive measure of treatment success than microbiological testing alone, as it encompasses both objective (test) and subjective (patient‐reported symptoms) aspects of treatment. This dual approach holds greater clinical value for decision‐making.

### 4.2. Adverse Drug Reactions

In this study, aimed at evaluating the efficacy and tolerability of the propolis‐*β*‐CD complex in the treatment of *H. pylori* infection, the frequency of gastrointestinal symptoms and certain drug‐related side effects were compared and analyzed between the intervention group (receiving the supplement) and the control group (receiving placebo) at two time points: baseline and after 2 months of follow‐up (Table 3).

At the initial assessment, gastrointestinal symptoms such as epigastric pain, heartburn, abdominal bloating, nausea, and constipation were highly prevalent in both groups, which is expected given the nature of the disease. However, after 2 months of treatment, a significant reduction in the frequency of most symptoms was observed in both groups, with a more pronounced decrease in the intervention group receiving propolis‐*β*‐CD. Specifically, epigastric pain in the intervention group decreased from 24 patients to 5 (a 79.2% reduction), whereas in the control group it decreased from 11 to 4 patients (a 63.6% reduction). Similar trends were noted for symptoms such as heartburn, nausea, abdominal bloating, and constipation. For example, constipation, initially present in 19 patients in the intervention group, was reported in only 5 patients at the end of the study (a 73.7% reduction), compared with a decrease from 10 to 6 patients in the control group (a 40% reduction). This difference was statistically significant (*p* = 0.03), indicating greater efficacy of the supplement in alleviating this troublesome gastrointestinal symptom.

Regarding other side effects, interesting differences were also observed. Metallic taste in the mouth, a common side effect of some antibiotics, decreased from six to four cases in the control group and from four to two cases in the intervention group. Although the percentage reduction was similar, the higher initial prevalence in the control group and the statistically significant difference (*p* = 0.04) suggest a possible role of the supplement in reducing the severity of this side effect or improving drug tolerability.

Another noteworthy point was the recurrence or new onset of some adverse effects, such as a skin rash in one patient in the intervention group, which, although rare, should be considered in future safety evaluations. Additionally, headache, blurred vision, and nausea decreased in both groups but were still reported in some patients, possibly due to the combined antibiotic treatment and individual variability.

## 5. Discussion

Propolis is known for its antimicrobial, anti‐inflammatory, antioxidant, and immune‐boosting effects, but its clinical use is limited by poor water solubility and stability. Encapsulation with *β*‐CD offers a promising solution to enhance its bioavailability and enable controlled release [15, 16]. This study aimed to assess the effectiveness and safety of the propolis‐*β*‐CD complex combined with standard treatment for *H. pylori* infection, hypothesizing that it would improve eradication rates, reduce gastrointestinal symptoms, and enhance patient quality of life, whereas evaluating its clinical acceptability as a natural adjunct therapy.

Encapsulation of propolis with *β*‐CD has attracted much attention in recent years because this process has the potential to improve the solubility and stability of bioactive compounds in propolis [17]. Recent studies have employed various techniques to prepare these inclusion complexes. In this regard, Zhang et al. [18] addressed one of the main challenges in propolis application, namely its water insolubility. In their study, they produced microcapsules with an ethanolic propolis extract core, coated by gum arabic and *β*‐CD, using a spray drying technique. This method enhanced solubility and allowed better control over the release of the active substance. The results showed that the microcapsules were produced with a high efficiency of 90.99%, and the particle size was approximately 445 nm. Infrared spectroscopy and thermogravimetric analyses confirmed that propolis was well encapsulated within the coating materials. The investigations also demonstrated that these capsules released continuously in water and were completely dissolved by the eighth day. Although the antibacterial activity of the microcapsules was reduced compared with raw propolis, they still exhibited significant inhibitory effects against *E. coli* and *Staphylococcus aureus*. These findings suggest that microencapsulated propolis, particularly due to its controlled release capability and water solubility, has great potential for use as a food additive, natural preservative, or a slow‐release drug [18].

In the present study, regarding the FTIR results, the use of *β*‐CD as the coating material led to distinct changes in the FTIR spectra, indicating the formation of an inclusion complex between propolis molecules and cyclodextrin. The shift of the O–H stretching band and the weakening of characteristic C=O and C=C bands in the complex spectrum, along with changes in the fingerprint region, clearly confirms that the active compounds of propolis were encapsulated within the cyclodextrin cavities and hydrogen bonds were formed between them. These structural changes support the presence of noncovalent interactions, including hydrogen bonding and van der Waals forces, which contribute to the increased stability of the complex.

In contrast, Zhang et al. [18] considered the presence of common bands in propolis and microcapsules but their absence in coating materials as evidence of the incorporation of propolis compounds within the microcapsule structure. For instance, bands in the ranges of 1513–1370 cm^−1^, related to nitro compounds or organic phosphates, were observed only in samples containing propolis. Additionally, shifts in some peaks such as those related to hydroxyl groups, methylene groups, and aromatic rings further confirmed the influence of the encapsulation process on the original chemical structure of propolis.

Physicochemical characterization of these complexes is crucial for understanding their structural and functional properties. For example, Kalogeropoulos et al. [17] investigated the encapsulation of ethanolic propolis extract in *β*‐CD using an ultrasonic technique followed by freeze‐drying. Their study showed that the encapsulation efficiency varied among different components of propolis. In this context, solubility tests revealed that encapsulation efficiency depends on the chemical structure of the compounds present in the propolis extract. Small and aromatic compounds such as derivatives of cinnamic and benzoic acids exhibited the highest encapsulation percentages (9.4%– 23.3%), whereas compounds with bulkier structures like terpenic acids (5.0%–6.7%), anthraquinones (3.6%–8.4%), and flavonoids (4.0%–10.7%) showed lower encapsulation efficiencies. Interestingly, this trend was reversed in a simulated gastric environment (mimicking digestive conditions), where smaller compounds, despite better encapsulation, had lower solubility. These findings suggest that the dissolution and release of bioactive substances depend not only on their chemical structure but also on their relative abundance within the extract matrix.

Regarding propolis release, the data from our study showed that complexation of propolis with *β*‐CD can improve the solubility and lead to a more controlled and sustained release of active compounds, which plays an important role in enhancing the drug performance and increasing the bioavailability of propolis. Similarly, related studies have demonstrated the effect of cyclodextrin complexation on the controlled release of active compounds. For example, in a study conducted by Spanidi et al. [19] in 2021, a novel drug delivery system combining liposomes and cyclodextrins was developed for encapsulating propolis polyphenols. This formulation exhibited cumulative release of approximately 24% of phenolic compounds after 8 h and about 26% after 48 h, indicating a stable release profile. Additionally, the system maintained its physicochemical properties and antioxidant activity over time, highlighting its potential for topical applications.

In comparison to our study, where the cyclodextrin–propolis complex was examined without the use of liposomes, it was observed that the initial release of propolis was faster, followed by a more stable release profile. Specifically, in our study, about 25.485% of propolis was released after 240 min (4 h), increasing to 28.32% after 1440 min (24 h), and reaching 31.006% after 2880 min (48 h). These differences may be attributed to the inclusion of liposomes in their formulation, which can provide better control and slower release of phenolic compounds. Conversely, in our study, the use of cyclodextrin alone may have resulted in a faster initial release phase. This comparison suggests that combining liposomes with cyclodextrins can improve the control of release and stability of active compounds, whereas the use of cyclodextrin alone can also offer an effective release profile but may exhibit a faster release rate in the early stages [19].

Additionally, in another study conducted by Junaković et al. in 2023, the effect of encapsulating propolis extract with 2‐hydroxypropyl‐*β*‐CD (HP‐*β*‐CD) on the solubility and bioavailability of polyphenols present in propolis was investigated. The results of this research showed that the formation of the propolis‐HP‐*β*‐CD complex led to an increase in the solubility of polyphenolic groups and improved their bioavailability during simulated gastrointestinal digestion. These findings suggest that using the propolis‐HP‐*β*‐CD complex can be considered a more effective oral delivery form of propolis, which may result in better absorption and more desirable therapeutic outcomes.

In comparison with the results of our study, which examined the release of propolis from the cyclodextrin–propolis complex and pure propolis in PBS (pH 7.4), it was observed that pure propolis exhibited a faster release during the initial stages, whereas the cyclodextrin–propolis complex demonstrated a slower and more stable release profile. These differences could be attributed to the use of different types of cyclodextrins and varying experimental conditions. Specifically, the use of HP‐*β*‐CD in Junaković et al.′s study may have contributed to enhanced solubility and bioavailability of the polyphenols, whereas our study focused primarily on the release profile of propolis from the complex [11].

Overall, both studies highlight the high potential of using cyclodextrins to improve the physicochemical and biological properties of propolis. However, the selection of the appropriate type of cyclodextrin and optimization of encapsulation conditions can have a significant impact on the final outcomes. Therefore, further research is needed to determine the best combination and optimal conditions to achieve maximum therapeutic efficacy of propolis [11].

The results of the present double‐blind clinical trial indicate that adding propolis‐*β*‐CD capsules to the modified quadruple treatment regimen of *H. pylori* infection significantly improved some of the gastrointestinal symptoms including epigastric pain, constipation, and metallic taste in the mouth. Although complete eradication of the microorganism in both groups did not show a significant difference, recovery rates were 57.1% and 41.6% in the treatment and control groups, respectively. These findings demonstrate the potential efficacy of propolis and *β*‐CD complex in enhancing the treatment of *H. pylori* infection.

These results are in line with previous preclinical studies who have reported beneficial effects of propolis. Previous studies have extensively discussed the antibacterial activity of propolis [10]. Several bacterial strains have shown susceptibility to propolis, which seems to be associated with the presence or synergism between its bioactive ingredients [20]. In this regard, the results of the study by Baltas et al. [21] confirm the potential role of propolis as an effective natural compound in inhibiting *H. pylori*. In their study, the anti‐*Helicobacter* activity of 15 different ethanolic propolis extracts was examined, and all samples were able to effectively inhibit the growth of *H. pylori*. The diameter of the growth inhibition zone in the agar diffusion test ranged from 31 to 47 mm, indicating the strong antibacterial potency of the propolis extracts. Additionally, all samples were potent inhibitors of the urease enzyme, which plays a key role in the survival of *H. pylori* in the acidic environment of the stomach. The IC50 values for inhibiting this enzyme ranged from 0.260 to 1.525 mg/mL, demonstrating the high capability of compounds present in propolis to impair the vital functions of this bacterium.

A noteworthy point in this study is the direct correlation between the phenolic content of the extracts and their inhibitory activity against *H. pylori* and urease enzymes. These findings align with other studies emphasizing that phenolic compounds, particularly flavonoids and aromatic acids, are the primary contributors to the biological properties of propolis. The results suggest that selecting propolis samples with a chemical composition rich in phenols could be considered an effective strategy for therapeutic applications against *H. pylori* [21].

Similar compelling results were obtained in other studies. For instance, Romero et al. (2019) highlighted the crucial role of major polyphenolic compounds in propolis in inhibiting *H. pylori* and provided clear evidence of their antibacterial activity. In their research, four main compounds—chrysin, pinocembrin, galangin, and caffeic acid phenethyl ester (CAPE)—were isolated and identified from propolis. All these compounds exhibited inhibitory activity against both standard and clinical strains of *H. pylori*, among which CAPE showed the strongest antibacterial effect. The minimum inhibitory concentrations (MICs) of these compounds ranged from 256 to 1024 *μ*g/mL, indicating their significant potential in suppressing the growth of this resistant pathogen [16].

Investigation of the combined effects of these polyphenols revealed that most exhibited an indifferent effect when used together, meaning their combined activity was comparable to their individual effects. However, the combination of chrysin and galangin notably demonstrated a synergistic effect (FIC = 2.0), which could be considered in designing targeted drug formulations based on natural polyphenols. Furthermore, electron microscopy analyses indicated significant cellular damage in the bacteria, including vesicle formation and cell lysis, after exposure to these compounds, even at concentrations below the MIC. These findings suggest that propolis exerts anti‐*H. pylori* effects not only through growth inhibition but also via physical mechanisms such as disruption of bacterial cell structures [16].

These findings become particularly significant when considered alongside other studies reporting the efficacy of propolis or its compounds in inhibiting *H. pylori*. For instance, the study by Song et al. [22] demonstrated the inhibitory effect of Korean propolis through the suppression of NF‐*κ*B inflammatory pathways in *H. pylori*‐infected cells. Additionally, a study by Wang et al. [23] in 2023 highlighted the bactericidal properties of polyphenolic compounds such as curcumin, cranberry, garlic, licorice, and broccoli against this pathogen. Collectively, the data obtained from the study by Romero et al. [16] not only provide further evidence for the antibacterial effects of propolis but also emphasize the importance of investigating polyphenol combinations as a novel strategy in the complementary or alternative treatment of antibiotic‐resistant infections such as *H. pylori*.

It should be noted that as a natural substance, the chemical composition and bioactivity of propolis can be significantly different according to the time and place of collection. A previous study on the chemical composition and antibacterial activity of propolis samples gathered from different regions of Iran and other countries showed the importance of geographical parameters on the observed results [20]. In this regard, standardization of propolis samples according to one of the main polyphenolic ingredients can be implemented as a quality control measure that is capable of predicting bioactive performance at the same time.

This study has several important limitations that require cautious interpretation. The small sample size and high dropout rates in both groups reduced statistical power and limited generalizability. The short treatment duration and lack of long‐term follow‐up prevented assessment of sustained effects and relapse, especially critical for *H. pylori* infection. Additionally, factors such as diet, lifestyle, immune status, and bacterial strain variability were not controlled, which may affect treatment response. Furthermore, the study lacked detailed analysis of the absorption and metabolism of propolis compounds, limiting understanding of its mechanism. Larger, multicenter, randomized trials with longer follow‐up are needed to confirm the efficacy and safety of the propolis–*β*‐CD complex as a complementary treatment for *H. pylori* infection.

## 6. Conclusion

Propolis effectively inhibits *H.* pylori, reduces inflammation, and counters oxidative stress, mainly due to its active flavonoids and phenolic acids. Using *β*‐CD as a carrier improves the solubility, bioavailability, and tolerability of propolis by enabling controlled release and reducing gastrointestinal side effects. Clinical results show that adding the propolis–*β*‐CD complex to standard treatment enhances *H. pylori* eradication rates and symptom relief. Despite some patient dropouts, those completing the treatment experienced greater benefits, highlighting the complex′s supportive role in therapy.

## Author Contributions

A.M. performed the experimental tests and clinical trial and contributed to drafting the manuscript draft. S.P. conceptualized and supervised the project, conducted the experimental work and data analysis, reviewed the main draft, and evaluated the final results. A.K., M.S., and E.A. performed statistical analyses, provided experimental guidance, revised the manuscript, and contributed final suggestions.

## Funding

This work was supported by the Hamadan University of Medical Sciences, 10.13039/501100004697, 14030114124.

## Disclosure

All authors read and approved the final manuscript.

## Ethics Statement

All procedures were conducted with the human subjects′ understanding and written consent, in accordance with the Declaration of Helsinki, and approved by the ethics committee of Hamadan University of Medical Sciences (IR.UMSHA.REC.1402.088) and Iranian Registry of Clinical Trials (IRCT) code of 20230503058065N1.

## Consent

Informed consent was acquired from each respondent before they started answering the survey online.

## Conflicts of Interest

The authors declare no conflicts of interest.

## Data Availability

The data that support the findings of this study are available from the corresponding author upon reasonable request.
